# Single CSTR can be as effective as an SBR in selecting PHA-storing biomass from municipal wastewater-derived feedstock

**DOI:** 10.1016/j.wroa.2023.100165

**Published:** 2023-01-10

**Authors:** Antoine Brison, Pierre Rossi, Nicolas Derlon

**Affiliations:** a Eawag, Swiss Federal Institute of Aquatic Science and Technology, 8600 Dübendorf, Switzerland; b ETH Zürich, Institute of Environmental Engineering, 8093 Zürich, Switzerland; c Central Environmental Laboratory, School of Architecture, Civil and Environmental Engineering, Ecole Polytechnique Fédérale de Lausanne Lausanne, Switzerland

**Keywords:** Polyhydroxyalkanoates, Nitrogen limitation, Microbial community analysis, Continuous-flow stirred-tank reactor, Aerobic feast-famine, Selection-step

## Abstract

A key step for the production of polyhydroxyalkanoates (PHAs) from organic waste streams is the selection of a biomass with a high PHA-storage capacity (selection-step), which is usually performed in sequencing batch reactors (SBR). A major advancement would be to perform such selection in continuous reactors to facilitate the full-scale implementation of PHA production from municipal wastewater (MWW)-derived feedstock. The present study therefore investigates to what extent a simple continuous-flow stirred-tank reactor (CSTR) represents a relevant alternative to anSBR. To this end, we operated two selection reactors (CSTR vs*.* SBR) on filtered primary sludge fermentate while performing a detailed analysis of the microbial communities, and monitoring PHA-storage over long-term (∼150 days) and during accumulation batches. Our study demonstrates that a simple CSTR is as effective as an SBR in selecting biomass with high PHA-storage capacity (up to 0.65 gPHA gVSS^−1^) while being 50% more efficient in terms of substrate to biomass conversion yields. We also show that such selection can occur on VFA-rich feedstock containing nitrogen (N) and phosphorus (P) in excess, whereas previously, selection of PHA-storing organisms in a single CSTR has only been studied under P limitation. We further found that microbial competition was mostly affected by nutrient availability (N and P) rather than by the reactor operation mode (CSTR vs. SBR). Similar microbial communities therefore developed in both selection reactors, while microbial communities were very different depending on N availability. *Rhodobacteraceae* gen. were most abundant when growth conditions were stable and N-limited, whereas dynamic N- (and P-) excess conditions favoured the selection of the known PHA-storer *Comamonas*, and led to the highest observed PHA-storage capacity. Overall, we demonstrate that biomass with high storage capacity can be selected in a simple CSTR on a wider range of feedstock than just P-limited ones.

## Highlights

• Selection of PHA-storing biomass on fermented municipal WW in a CSTR was studied.
• Similar PHA-storage capacity and microbial communities in CSTR vs. feast-famine SBR.
• CSTR more efficient in terms of substrate to biomass conversion yields than SBR.
• High PHA content and selection of *Comamonas* sp. under excess of P and N.
• Low PHA content and selection of *unknown Rhodobacteraceae* sp. under N-limitation.

## Graphical abstract

**Figure:**
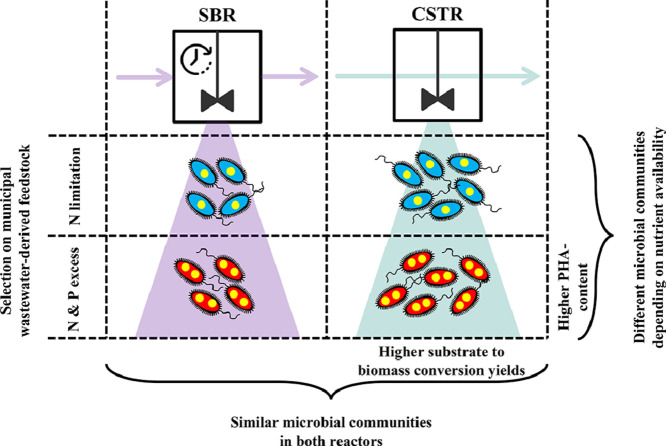

## 1 Introduction

The recovery of organic carbon (C) from municipal wastewater (MWW) in the form of polyhydroxyalkanoates (PHAs) (bioplastics) is more sustainable than its conversion into energy *via* biogas production (Hao et al., 2022). A crucial step is however to select a biomass with a high PHA-storage capacity (selection-step), after the capture of the organic C of the MWW and its conversion into volatile fatty acids (VFAs) via fermentation (Alloul et al., 2018). The state-of-the-art approach is to perform this selection-step in a sequencing batch reactor (SBR) (discontinuous operation) (Estévez-Alonso et al., 2021; Valentino et al., 2017), while wastewater treatment plants (WWTPs) mostly operate in a continuous flow mode, e.g., anaerobic fermenters. The discontinuous nature of the SBR brings inconveniences – such as steep gradients in oxygen requirements throughout the cycle, as well as the need for high buffer volumes – which could be avoided by using continuous systems (Marang et al., 2015). A relevant opportunity to facilitate the implementation of PHA production units at WWTPs is therefore to perform the selection-step a single continuous-flow stirred-tank reactor (CSTR) that could be integrated more easily to existing facilities already operating in a continuous-flow mode. However, to date, the selection of PHA-storing biomass in a single CSTR remains poorly understood while it has never been demonstrated for MWW-derived feedstock.

The selection of PHA-storing biomass on waste-derived feedstock is usually performed in SBRs operated in an aerobic feast-famine mode (SBR-approach) (Estévez-Alonso et al., 2021; Valentino et al., 2017), where the biomass is sequentially exposed to presence/absence of exogenous C-substrate. A main competitive advantage of PHA-storing organisms (PHA-storers) over non-storing organisms is their ability to store PHA under feast conditions, and then to utilize such C source for growth during the following famine period (Reis et al., 2011). It is therefore key that growth essential nutrients, such as nitrogen (N) and phosphorus (P), are available during the famine period so that PHA-storers can use the stored PHA for growth. Feedstocks characterized by an excess of these nutrients are thus particularly suitable for the selection of PHA-storers based on the SBR approach. On the contrary, a limited availability of those nutrients will inevitably hamper the selection of PHA-storers (Albuquerque et al., 2007; Johnson et al., 2010; Korkakaki et al., 2017) while the availability of N and P greatly varies in MWW-derived feedstock. Dissolved COD:N (COD = chemical oxygen demand) and COD:P ratios in the range of 5–70 gCOD gN^−1^ and 38–1000 gCOD gP^−1^ are reported in literature (Brison et al., 2022b; Conca et al., 2020; Da Ros et al., 2020; Soares et al., 2010; Ucisik and Henze, 2008), whereas nutrient limitation potentially starts at COD:N and COD:P ratios >25 gCOD gN^−1^ and/or >140 gCOD gP^−1^, respectively (Tchobanoglous et al., 2013).^1^ While such feedstock composition might be detrimental to the selection process in a SBR, nutrient limitation might actually offer a relevant opportunity to select PHA-storing biomass in a single CSTR.

In natural aquatic environments associated with nutrient-limited conditions, PHA-storage provides a competitive advantage to storing microorganisms (Ovreas et al., 2003; Thingstad et al., 2005). In engineered systems, PHA-storage has for example been observed for high COD:N/P ratios in the influent (Anderson and Dawes, 1990; Paul et al., 2020). Using a simple synthetic influent (100% acetate), Cavaille et al. (2016) reported a PHA content increase from 0.2 to 0.8 gPHA gVSS^−1^ in the biomass of a CSTR exposed to a dynamic increase of the influent COD:P ratio (∼400–3000 gCOD gP^−1^) and SRT (1–2 days). Further, Brison et al. (2022a) were able to select a stable microbial community consisting of >90% PHA-storers at a constant influent COD:P ratio of 800 gCOD gP^−1^, while using a synthetic influent more representative of MWW-derived feedstocks (50% acetate-propionate). But our current knowledge in selecting PHA-storers in continuous systems is limited to the use of synthetic influents with acetate/propionate as the sole C-substrates, while MWW-derived feedstock often contains a complex mix of VFAs (acetate, propionate, (iso)-butyrate, (iso)-valerate) as well as non-VFA substrate, which can make up to 50% of the soluble COD (Morgan-Sagastume et al., 2011, 2014). Also, investigations were limited to high influent COD:P ratios while nutrients other than P (e.g., N) can be limiting in MWW-derived feedstock (Brison et al., 2022b; Da Ros et al., 2020). Finally, the composition of MWW-derived feedstock (e.g., the COD:N/P ratio) might be subject to natural temporal dynamics, while dynamic environmental growth conditions, such as in WWTPs, tend to provide ecological niches to PHA-storers (Pei et al., 2022). While previous studies using synthetic wastewater provided strong evidence that a simple CSTR can be used to select PHA-storers, it is now essential to evaluate to what extent such reactor operation mode also works on real municipal WW.

The present study aims, for the first time, at investigating to what extent a simple CSTR can be successfully used to select a biomass with a high PHA-storage capacity on a MWW-derived feedstock, as opposed to a state-of-the-art SBR. The specific following questions were addressed: (i) How does the reactor operation mode (SBR vs*.* CSTR) influence the selected microbial communities, and in turn the PHA-storage capacity of the biomass, and (ii) To what extent is microbial competition and the selection of PHA-storers influenced by nutrient (N and P) availability when using real MWW-derived feedstock? To answer these questions, an SBR (aerobic feast-famine mode) and a CSTR were fed with the filtered effluent of a primary sludge fermenter. Microbial communities, PHA-storage, substrate to biomass conversion yields, as well as growth conditions with respect to NH_4_^+^-N and PO_4_^3−^-P were monitored over long-term (∼150 SRTs). In addition, several PHA-accumulation tests were carried out with the selected biomasses.

## 2 Materials and methods

### 2.1 Overall experimental approach

**Fig. 1:**
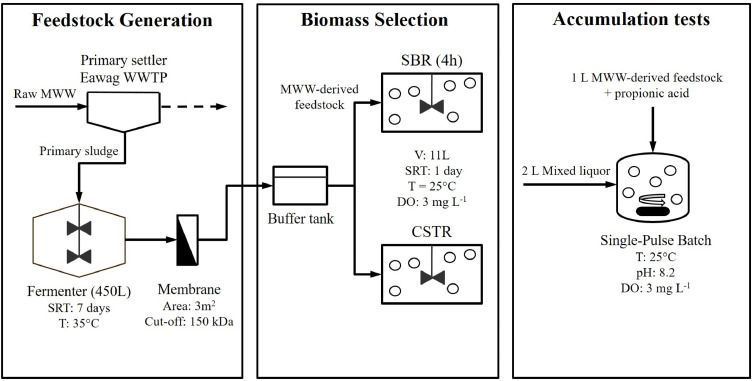
Overall experimental set-up with the different sub-units and most important operating conditions.

Two reactors, an SBR (aerobic feast-famine mode) and a CSTR, were operated in parallel as selection reactors over an extended period of ∼150 days and fed with MWW-derived feedstock. Both reactors had a working volume of 11 L. Several accumulation tests were performed to assess the PHA-storage capacity of the selected biomasses. The overall experimental approach thus consisted of three sub-systems: (i) production of MWW-derived feedstock, (ii) selection of PHA-storing biomass, and (iii) testing the PHA-storage capacity of the selected biomass (Fig. 1).

### 2.2 Detailed set-up and reactor operating conditions

#### 2.2.1 Production of MWW-derived feedstock

A continuously mixed 450 L fermenter (35 °C, 7 days SRT=HRT) was fed with primary sludge from the Eawag municipal WWTP to produce the MWW-derived feedstock. The fermenter effluent was pre-settled in a secondary clarifier and then filtered through a membrane module (Microclear MCXL, Newterra, Germany) consisting of 9 double-sided layers of 150 kDA polyethersulfone ultrafiltration membranes (Microdyn-Nadir, Germany) and operated in outside-inside mode. The total membrane surface of the module was 3 m^2^. Peristaltic pumps (530-IP31, Watson-marlow, Switzerland) were connected at both outlets of the membrane module. Filtration was performed in six daily cycles of 2 h at an average flow of 1.5 l m ^−^ ^2^ h ^−^ ^1^. The membrane was backwashed twice a day for 15 min with the filtered permeate at a similar flux. The filtered permeate was temporarily stored in a buffer tank before being fed to the selection reactors. The storage time in the buffer tank was maximum 1 d, while oxygen transfer was prevented with floating balls.

#### 2.2.2 Selection of PHA-storing biomass

Double-wall glass reactors with a working volume of 11 L were operated in SBR or CSTR mode at an SRT (= HRT) of 1 day, in order to maximize the substrate-to-biomass conversion yield. The SBR was operated in aerobic feast-famine mode. The cycle duration and volume exchange ratio were 4 h and 0.17, respectively. The cycle consisted of 3 phases: (i) 5 min of feeding (flow rate: 0.37 L min^−1^), (ii) 230 min of reaction at constant volume, and (iii) 5 min of withdrawal. Aeration and mixing were active during all 3 phases. SRT was controlled by purging mixed liquor at the end of the cycle (i.e., 1.83 L) without prior sedimentation nor supernatant decanting. The CSTR was fed at an average flow-rate of 7 mL min^−1^). Both selection reactors were equipped with temperature, dissolved oxygen (DO) and pH sensors (Endress & Hauser, Switzerland). Sensors were connected to a programmable logic controller (PLC) and monitored by a supervisory control and data acquisition (SCADA) system. In both reactors temperature was controlled at 25 ± 1 °C, while DO was controlled at a set-point of 3 mg L ^−^ ^1^. A mechanical stirrer ensured fully mixed conditions (rotation speed: 160 rpm). The reactors were cleaned 2–3 times a week to remove biofilm from the walls. The MWW-derived feedstock was supplied to the selection reactors from the buffer tank using peristaltic pumps (300 series pumpheads, Watson-Marlow, Switzerland). The reactors were inoculated with high-rate activated sludge since such systems select microorganisms (i) with a high affinity towards intracellular storage of organic C, and (ii) at similarly low SRTs (0.1–2 days) than the one applied in our experiment (Jimenez et al., 2015; Nogaj et al., 2019; Sancho et al., 2019).

Samples for the analysis of the microbial communities, chemical parameters (COD, N and P species) and biomass PHA content were taken twice a week. SBR-samples were collected at the end of the feast phase (all parameters) and at the end of the cycle (only COD and PHA). The PHA-content of SBR biomass is subject to dynamics throughout the cycle and maximum PHA-content is observed at the end of the feast-phase (when all of the external C substrate is depleted). In contrast, the PHA-content observed in a CSTR is always at its maximum since no substrate gradients occur. Therefore, when comparing both selection reactors in terms of PHA-storage, we considered the SBR-samples from the end of the feast-phase. The end of the feast-phase was deduced from the DO and pH profiles, as explained in SI Fig. A1. The feast-phase lasted on average for 35 ± 15% of the cycle duration.

#### 2.2.3 PHA-accumulation tests

PHA-accumulation tests were conducted in double-wall glass reactors (5 L, Schmizo AG, Switzerland). The reactors were equipped with pH, temperature and DO sensors, all connected to a PLC and monitored by a SCADA system. Mixing was ensured through magnetic stirrers. The same temperature and DO control was applied as for the selection reactors. In addition, pH was controlled at the average value observed in the selection reactors (∼8.2) through automated addition of a 3 M HCl solution.

PHA-accumulation tests were conducted on biomass from both selection reactors on days 108, 115, 136 and 143. The accumulation tests consisted of 3 L batches with a single initial pulse of soluble C substrate. 2 L of mixed liquor from the selection reactor were mixed with 1 L of MWW-derived feedstock (same influent than for the selection reactors) to establish a similar bulk matrix (e.g., in terms of trace elements) to which the biomass was acclimatised. In order to ensure an excess of C substrate, the dissolved substrate concentration in the feedstock was previously increased fivefold by adding pure propionic acid (99.5%, Merck, Germany). Propionic acid was selected over other VFAs as it represented the main VFA fraction in the MWW-derived feedstock. Samples for analysis (PHA, VFA and NH_4_^+^-N) were taken every on an hourly basis. The batches were stopped after 12 h.

### 2.3 Influent feedstock composition

The influent feedstock composition (COD, VFAs, N and P species, microbial communities) was analysed every 4–5 days (Table 1). Influent COD in the dissolved form was >90% of the total COD, while VFAs represented ∼85% of the soluble COD (sCOD). Propionic acid was abundant and accounted for ∼80% of the VFAs in the feedstock. Overall, the feedstock was rather scarce in N, with sCOD:NH_4_^+^-N ratios >80 gCOD gN^−1^. The high standard deviations of individual parameters result from the natural variability in (i) the composition of the solids fed to the fermenter and (ii) of their concentration.

**Table 1:** Key parameters concerning the composition of the MWW-derived feedstock to the selection reactors. Average values ± standard deviation (number of measurements).

Parameter	Unit	Value
COD	mgCOD L ^−^ ^1^	3038 ± 1406 (28)
Soluble COD (sCOD) fraction	(-)	0.92 ± 0.04 (28)
VFA: sCOD ratio	(-)	0.85 ± 0.09 (28)
Propionate: VFA ratio	(-)	0.79 ± 0.04 (28)
sCOD: NH_4_^+^-N ratio	(gCOD gN^−1^)	84 ± 91 (28)
sCOD: PO_4_^3−^-P ratio	(gCOD gP^−1^)	120 ± 23 (28)
NH_4_^+^-N: PO_4_^3−^-P ratio	(gN gP^−1^)	2.2 ± 1.0 (28)

### 2.4 Analytical methods

#### 2.4.1 Chemical analyses

Samples were analysed for total COD, sCOD, total phosphorus (TP), ortho-phosphate (PO_4_-P), total nitrogen (TN) and ammonium nitrogen (NH_4__—_N) using colometric assays (Hach-Lange, Germany, LCK 014, 114, 303, 304, 338, 349, 350). Soluble COD (sCOD), NH_4__—_N and PO_4_-P were measured after filtration at 0.45 µm (Nanoclor Chromafil membranefilter GF/PET 0.45 µm, Macherey Nagel, Germany). Particulate COD (pCOD) was calculated by subtracting the measured sCOD from the measured total COD. VFAs were measured after filtration at 0.45 µm *via* ion chromatography (930 Compact IC Flex, Metrohm, Switzerland) using Metrosep Organic Acids 250/7.8 and Guard 4/6 columns (both Metrohm, Switzerland).

#### 2.4.2 PHA measurements

Biomass samples for PHA measurements were taken approximately twice a week. Samples were immediately frozen in liquid nitrogen to stop any biological activity, then stored at −18 °C prior lyophilisation. The lyophilised solids were then analysed for the most common PHA monomers produced by mixed microbial cultures from VFAs: 3HB, 3HV, 3H2MB and 3H2MV. PHA extraction, hydrolysis and analysis was performed according to Lanham et al. (2013) as described in the Supporting Information. A PHB-PHV co-polymer (86:14 wt%, Sigma-Aldrich) was used as a standard for 3HB and 3HV monomers. Industrial 3‑hydroxy-2-methylbutanoic and 3‑hydroxy-2-mehylpentanoic acids (both from Merck, Germany) were used as standards for 3H2MB and 3H2MV monomers, respectively. Since neither 3H2MB nor 3H2MV was detected in any of the analysed samples, the total PHA concentration was calculated as the sum of PHB and PHV concentrations, deduced from the measured 3HB and 3HV signals. Results were expressed on a COD basis (gCOD_PHA_ gpCOD^−1^) by using conversion factors of 1.67 gCOD gVSS_PHB_^−1^ and 1.92 gCOD gVSS_PHV_^−1^. For comparison with literature, results were also expressed in gPHA gVSS^−1^ by assuming a conversion factor of 1.42 gCOD gVSS^−1^ for non-PHA pCOD/solids.

#### 2.4.3 Microbial community analysis

Biomass samples were collected twice a week for 16 s rDNA gene sequencing. 1.5 mL of sludge were centrifuged at 12′000 × g for 5 min and washed twice in 3–4 mL of ice-cold phosphate saline buffer (PBS). The pellets were homogenized with a glass homogenizer, and stored at −80 °C until DNA extraction. DNA extraction was carried out as described in Layer et al. (2019) (Supporting Information A). Bacterial 16S rRNA gene hypervariable regions V1–V2 were amplified in a T3000 Thermocycler (Biometra, Germany) using 27F and 338R universal primers with overhang adapters (5′ TCGTCGGCAGCGTCAGATGTGTATAAGAGACAG-AGMGTTYGATYMTGGCTCAG3′) and (5′GTCTCGTGGGCTCGGAGATGTGTATAAGAGACA-GGCTGCCTCCCGTAGGAGT3′) (Layer et al., 2019). Amplification products were quantified on a Fragment Analyzer System with a NGS fragment kit (both Agilent, USA) prior to sequencing at the Lausanne Genomic Technologies Facility (University of Lausanne, Switzerland). Multiplex paired-end sequencing (2 × 250 bp) was carried out on an Illumina MiSeq platform. The raw sequences are accessible under https://doi.org/10.25678/0007SV.

The definition of OTUs and taxonomic affiliation was performed using the FROGS pipeline (Escudie et al., 2018; Poirier et al., 2018). OTUs containing less than 0.01% of all sequences were excluded, leaving a total of 716 OTUs. The reads per sample were on average ∼40′000.Taxons were affiliated using 16S Silva 138 (Quast et al., 2013). The BLAST tool of the MiDAS Field Guide (https://www.midasfieldguide.org/guide/blast) was subsequently used to improve/verfiy affiliation at the family/genus taxonomic level of the 150 most abundant OTUs. The generated output including changes according to the MiDAS data base is provided under https://doi.org/10.25678/0007SV. The freeware R version 4.0.2 (R Core Team, 2020) running on RStudio (version 1.3.1093) was used for numerical ecology analysis (Coral et al., 2018). Principal Component Analysis (PCA) and Redundancy Analysis (RDA) were carried out on the Hellinger transformed microbial data set using the Vegan package (Oksanen et al., 2020). Heatmaps were generated using Spearman pairwise correlations between environmental and microbial data sets. Detailed microbial community analysis (PCA, RDA, correlations and heatmaps) was performed at the Family taxonomic level to minimize the amount of multi- and non-affiliations. In addition, we selectively considered the Genus taxonomic level to discuss the presence of putative PHA-storers in our experimental systems, and to estimate, when possible, a minimum fraction of PHA-storers in the selected microbial community. However, taxonomic affiliation at the Genus level in this study did not allow for a holistic estimation of the fraction of PHA-storers as performed by Brison et al. (2022a).

### 2.5 Calculations

The selection reactors were compared in terms of biomass PHA content, HV:HB ratio, and substrate to biomass conversion yields. The biomass PHA content (gCOD_PHA_ gpCOD^−1^) was calculated as:

(1) $$\text{PHA}\;\text{content}\;=\;\frac{\text{CO}D_{\text{PHA}}}{\text{pCOD}}$$

With pCOD the particulate COD concentration (gCOD L ^−^ ^1^), and COD_PHA_ the PHA concentration in the reactor (gCOD L ^−^ ^1^).

The HV:HB ratio (-) was calculated on a COD basis as:

(2) $$\text{HV}:\text{HB}\;\text{ratio}\;=\;\frac{\text{CO}D_{\text{PHV}}}{\text{CO}D_{\text{PHB}}}$$

Where COD_PHV_ and COD_PHB_ were the measured PHV and PHB concentrations in the reactor (gCOD L ^−^ ^1^), respectively.

The substrate to active biomass conversion yield (Observed Yield, gCOD_Xact_ gsCOD_removed_^−1^) was calculated assuming no other solids than active biomass and PHAs were produced:

(3) $$\text{Observed}\;\text{Yield}\;=\;\frac{\left(\text{pCOD}-\text{CO}D_{\text{PHA}}\right)}{(\text{sCO}D_{\text{in}}-\text{sCOD})}$$

With sCOD_in_ the influent soluble COD concentration (gCOD L ^−^ ^1^), and sCOD the soluble COD concentration in the selection reactor (gCOD L ^−^ ^1^).

Finally, the VFA to PHA conversion yield (PHA yield, gCOD_PHA_ gCOD_VFA_^−1^) during accumulation batches was calculated as:

(4) $$\text{PHA}\;\text{Yield}\;=\;\frac{\left(\text{CO}D_{\text{PHA},\text{End}}-\text{CO}D_{\text{PHA},\text{Start}}\right)}{\left(\text{CO}D_{\text{VFA},\text{Start}}-\text{CO}D_{\text{VFA},\text{End}}\right)}$$

With COD_PHA,Start_ and COD_VFA,Start_ the PHA and VFA concentrations (gCOD L ^−^ ^1^) at the start of the batch, and COD_PHA,End_ and COD_VFA,End_ the PHA and VFA concentrations (gCOD L ^−^ ^1^) at the point in time when the PHA content stabilized.

The raw data of all measured parameters is accessible under https://doi.org/10.25678/0007SV.

## 3 Results

### 3.1 How does reactor operation mode (CSTR vs. SBR) influence the composition of the selected microbial community?

**Fig. 2:**
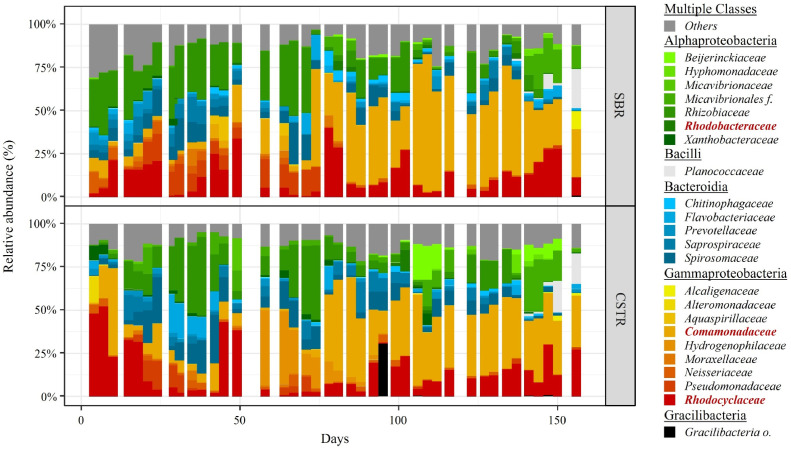
Microbial community composition over time in both selection reactors at the Family taxonomic level, and grouped at the Class taxonomic level. The group “Others” contains all taxa with a maximum relative abundance <8% with respect to all samples. The name of the three dominant families in the legend are highlighted in bold red.

**Fig. 3:**
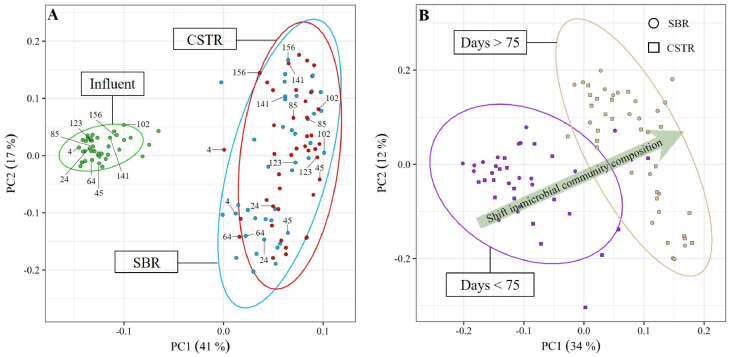
Principal Component Analysis (PCA) plots based on Hellinger transformed relative abundances of the bacterial taxa (Family level) present in both reactors (and the influent). Samples (dots) appearing close to each other can be expected to be similar in terms microbial community composition. Only taxa with a maximum relative abundance >8% with respect to all samples were considered. The ellipses cover the 90% confidence interval for each group of samples (as labeled on the respective plots), assuming a normal distribution. Thus, the ellipses are mainly a visual help to compare different clusters in terms of similarity of microbial community composition. (A) Samples are grouped by sample-location, i.e., Influent, SBR and CSTR. (B) Samples from the SBR (circles) and the CSTR (squares) were grouped chronologically according to the first half (Days < 75) and the second half (Days > 75) of the experiment.

The microbial communities in both selection reactors were monitored throughout the experiment (Fig. 2). Overall, the reactor operation mode had little to no influence on the microbial community composition. The CSTR and the SBR were therefore characterised by similar microbial communities, as shown by the analysis of both their composition and diversity (Fig. 3A, SI Fig. A2). *Comamonadaceae* gen., *Rhodobacteraceae* gen. and *Rhodocyclaceae* gen. were the most abundant taxa in both reactors, accounting on average for 57 ± 27% and 50 ± 26% of the sequences in the SBR and CSTR, respectively. The microbial communities selected in both reactors were very different from the microbial communities in the influent feedstock (Fig. 3A), indicating that the similar microbial communities observed in the SBR and CSTR did not result from a process of microbial immigration from the influent.

In terms of dynamics, a clear shift in the microbial community composition was observed in both selection reactors at around day 75 of the experiment (Fig. 3B). The microbial communities were indeed dominated by *Rhodobacteraceae* gen. in the 1st half (days < 75) of the experiment, whereas *Commamonadaceae* gen. dominated in the 2nd half (days > 75) of the experiment. The relative abundances of *Comamonadaceae* gen. and *Rhodobacteraceae* gen. therefor significantly changed between the 1st and the 2nd half of the experiment. The relative abundance of *Rhodobacteraceae* gen. decreased from 26 ± 10% (SBR) and 20 ± 15% (CSTR) in the 1st half of the experiment, to 8 ± 8% (SBR) and 6 ± 6% (CSTR) in the 2nd half of the experiment. In contrast, the relative abundance of *Comamonadaceae* gen. increased from 9 ± 13% (SBR) and 9 ± 11% (CSTR) in the 1st half of the experiment, to 43 ± 16% (SBR) and 36 ± 10% (CSTR) in the 2nd half of the experiment. In contrast, the relative abundance of *Rhodocyclaceae* gen. did not significantly change between the 1st and 2nd half of the experiment, and was on average 13 ± 10% (SBR) and 14 ± 14% (CSTR).

Considering the Genus taxonomic level, the most dominant taxa among the selected *Comamonadaceae* gen., *Rhodocyclaceae* gen. and *Rhodobacteraceae* gen. were *Comamonas* sp., *Zoogloea* sp. and an *unknown Rhodobacteraceae* sp., respectively (SI Fig. A3). *Comamonas* sp. accounted on average for 35 ± 21% (SBR) and 31 ± 12% (CSTR) of the sequences during the 2nd half of the experiment. The relative abundance of *Zoogloea* sp. was 2% ± 2% (SBR) and 9 ± 12% (CSTR) during the 1st half of the experiment, and 13% ± 9% (SBR) and 11 ± 8% (CSTR) during the 2nd half of the experiment. The *unknown Rhodobacteraceae* sp. accounted on average for 24 ± 11% (SBR) and 16 ± 16% (CSTR) of the sequences during the 1st half of the experiment.

### 3.2 How does reactor operation mode influence PHA-storage and substrate to biomass conversion yields in the selection reactors?

**Fig. 4:**
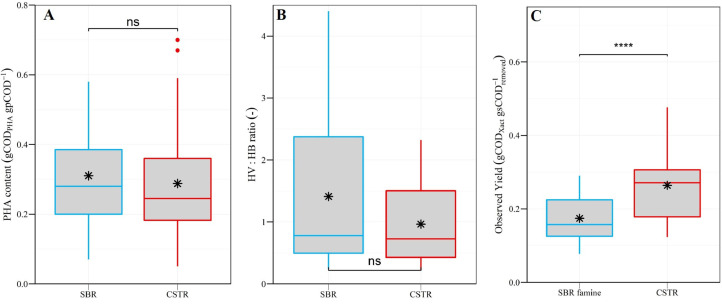
Box-plots presenting the (A) PHA content, (B) HV:HB ratio and (C) substrate to biomass conversion yield observed throughout the experiment in both selection reactors. For the SBR, PHA content and HV:HB ratio are shown for the end of the feast-phase (“SBR”), while observed yields are shown for the end of the famine-phase (“SBR famine”) (end of cycle). The black stars in the boxes are the mean values. The symbols on top of the brackets indicate the statistical significance of the difference between mean values according to an Independent Samples *t*-test: ns (not significant, *p* ≥ 0.05), * (*p*<0.05), ** (*p*<0.01), *** (*p*<0.001) and **** (*p*<0.0001).

**Fig. 5:**
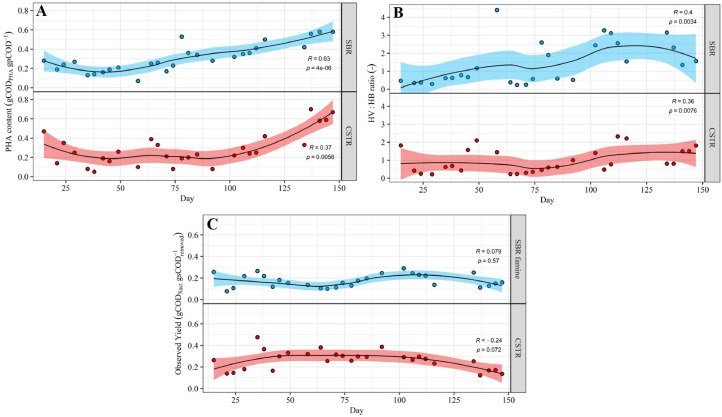
Time series of (A) biomass PHA content (COD basis), (B) HV:HB ratio and (C) substrate to biomass conversion yields in both selection reactors. For the SBR, PHA content and HV:HB ratio are shown for the end of the feast-phase (“SBR”), while observed yields are shown for the end of the famine-phase (“SBR famine”) (end of cycle). Local regression fitting (LOESS) (black line) and 95% confidence interval (shaded area) were added to help visualize general trends. The Kendall's tau coefficient (R) indicates whether the variable of interest followed a strong monotonic upward (*R*>0.3) or downward (*R*<−0.3) trend over time. The p-value (p) indicates the statistical significance of the observed trend. Only p-values < 0.05 were considered statistically significant.

Since similar microbial communities were selected in the CSTR and SBR, an important question is to what extent both selection reactors differed in terms of biomass PHA content, PHA composition and substrate to biomass conversion yields (Figs. 4A-C and 5A-C). Over the entire experiment, the CSTR was more efficient than the SBR in terms of substrate to active biomass conversion, while no difference was observed between both reactors in terms of PHA-storage (Fig. 4A-C). Similar mean PHA contents of 0.29 ± 0.18 gCOD_PHA_ gpCOD^−1^ (resp. 0.25 ± 0.15 gPHA gVSS^−1^) and of 0.31 ± 0.14 gCOD_PHA_ gpCOD^−1^ (resp. 0.26 ± 0.11 gPHA gVSS^−1^) were measured over the whole experiment for the CSTR and for the SBR, respectively. Also, no statistically significant difference was observed between the mean HV:HB ratio in the CSTR (1.0 ± 0.7) and the one in the SBR (1.4 ± 1.2). However, substrate to biomass conversion yields were much higher in the CSTR as opposed to the SBR: 0.26 ± 0.09 vs. 0.17 ± 0.06 gCOD_Xact_ gsCOD_removed_^−1^, respectively.

Similarily to the microbial community composition, the PHA storage also changed significantly over the course of the experiment. In both selection reactors the PHA content was thus significantly higher in the 2nd half (days > 75) compared to the 1st half (days < 75) of the experiment (Fig. 5A). More specifically, the PHA content varied around an average value of 0.2 gCOD_PHA_ gpCOD^−1^ during the 1st half of the experiment, but then gradually increased up to 0.7 gCOD_PHA_ gpCOD^−1^ (0.65 gPHA gVSS^−1^) and 0.6 gCOD_PHA_ gpCOD^−1^ (resp. 0.54 gPHA gVSS^−1^) in the CSTR and SBR respectively, during the 2nd half of the experiment. Further, the HV:HB ratios gently increased throughout the experiment: from ∼1.0 to ∼1.5 and from ∼0.5 to ∼2.0 in the CSTR and the SBR, respectively (Fig. 5B). Finally, substrate to biomass conversion yields remained relatively stable throughout the experiment, as indicated by local regression and Kendall's tau coefficients (Fig. 5C).

### 3.3 How does nutrient availability influence microbial selection and PHA-storage in the selection reactors?

**Fig. 6:**
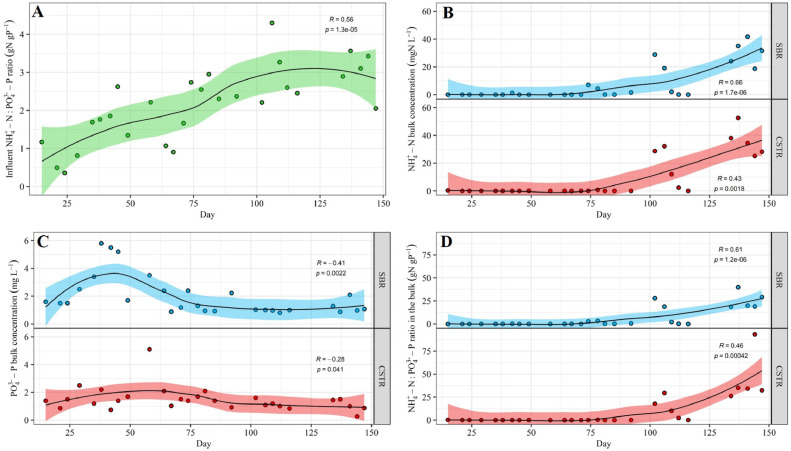
Time series of (A) NH_4_^+^-N: PO_4_^3−^-P ratio in the influent, and (B) NH_4_^+^-N concentrations, (C) PO_4_^3−^-P concentrations and (D) NH_4_^+^-N: PO_4_^3−^-P ratio in the bulk of the selection reactors. For the SBR, parameters were measured/calculated based on samples by the end of the feast-phase. Local regression fitting (LOESS) (black line) and 95% confidence interval (shaded area) were added to help visualize general trends. The Kendall's tau coefficient (R) indicates whether the variable of interest followed a strong monotonic upward (*R*>0.3) or downward (*R*<−0.3) trend over time. The p-value (p) indicates the statistical significance of the observed trend. Only p-values < 0.05 were considered statistically significant.

Nutrient availability was monitored in the influent and in both selection reactors throughout the experiment (Fig. 6A-D, SI Fig. A4 A-B). The influent N availability relative to P and C-substrate (sCOD) increased throughout the experiment (Fig. 6A, SI Fig. A4 A). The NH_4_^+^-N:PO_4_^3−^-P ratio in particular tripled from 1 to 3 gN gP^−1^.

As N availability increased in the influent, N availability in the selection reactors also underwent similar temporal dynamics for both CSTR and SBR (Fig. 6B). NH_4_^+^-N bulk concentrations remained mostly < 0.1 mgN L ^−^ ^1^ during the 1st half of the experiment, but then significantly increased to >40 mgN L ^−^ ^1^ during the 2nd half of the experiment. While NH_4_^+^-N bulk concentrations increased by more than two orders of magnitude during the 2nd half of the experiment, PO_4_^3−^-P bulk concentrations remained relatively stable between 1 and 2 mgP L ^−^ ^1^ (Fig. 6C). Consequently, NH_4_^+^-N:PO_4_^3−^-P ratios in the bulk followed the same temporal pattern than NH_4_^+^-N concentrations: very low and stable values (<< 0.1 gN gP^−1^) in the 1st half of the experiment, followed by a gradual increase in the 2nd half of the experiment to values between 25 and 40 gN gP^−1^ (Fig. 6D). In terms of growth conditions, NH_4_^+^-N concentrations < 0.1 mgN L ^−^ ^1^ together with NH_4_^+^-N:PO_4_^3−^-P ratios << 0.1 gN gP^−1^ suggest N was the limiting nutrient during the 1st half of the experiment. During the 2nd half of the experiment, neither N nor P may have limited growth, as NH_4_^+^-N concentrations increased dramatically, and PO_4_^3−^-P concentrations ranged between 1 and 2 mgP L ^−^ ^1^. However, the increasing NH_4_^+^-N:PO_4_^3−^-P ratio in the bulk means that the availability of P relative to N was decreasing.

**Fig. 7:**
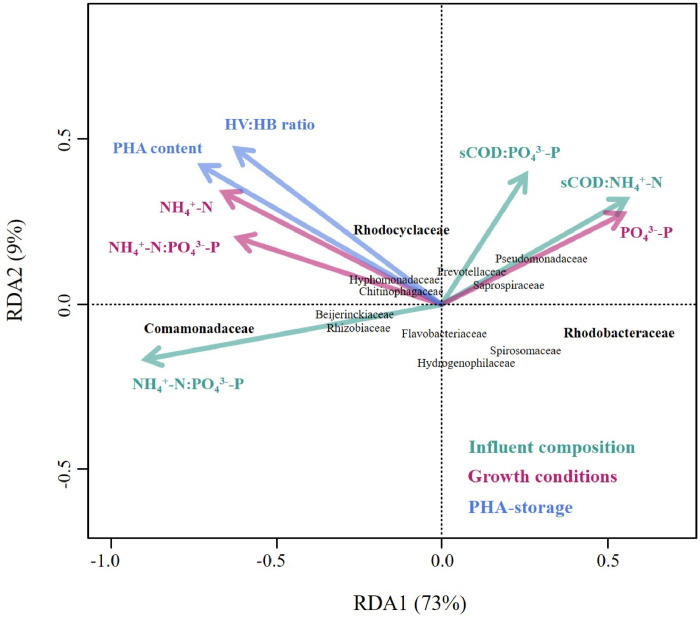
RDA-plot showing the relationship between environmental variables and the bacterial taxa at the Family taxonomic level. The environmental variables are grouped in three categories. (i) Influent composition with respect to nutrient availability (dark teal arrows) in terms of sCOD:PO_4_^3−^-P, sCOD:NH_4_^+^-N and NH_4_^+^-N: PO_4_^3−^-P ratios (dark teal vectors). (ii) Growth conditions with respect to nutrient availability in terms of NH_4_^+^-N:PO_4_^3−^-P ratio as well as NH_4_^+^-N and PO_4_^3−^-P concentrations in the reactor (pink vectors). (iii) PHA-storage in the selection reactor with respect to PHA content and HV:HB ratio (blue vectors). Phenomenologically, influent composition and growth conditions should be viewed as factors influencing the microbial community composition, while PHA-storage should be viewed as a factor being influenced by the microbial community composition. The plot is shown in scaling 2, where the angle between vectors indicates the similarity between environmental variables in terms of their correlation with the individual bacterial taxa. Because RDA1 explains 73% of the system's variance, scattering along the horizontal axis expresses more significant relationships than scattering along the vertical axis.

Statistical relationships between (i) influent nutrient availability, (ii) nutrient availability in the reactors, (iii) microbial community composition and, (iv) PHA-storage, were studied for a combined data-set due to the similarity between CSTR and SBR with respect to the above-mentioned variables (Fig. 7, SI Fig. A5, SI Table A1). The influent NH_4_^+^-N:PO_4_^3−^-P ratio showed a strong positive correlation with NH_4_^+^-N concentrations and NH_4_^+^-N:PO_4_^3−^-P ratios in the bulk (SI Fig. A5). These three variables all correlated (i) positively with *Comamonadaceae* gen., and (ii) negatively with *Rhodobacteraceae* gen. (Fig. 7, SI Table A1). In contrast, the relative abundance of *Rhodocyclaceae* gen. was only weakly influenced by NH_4_^+^-N concentrations and NH_4_^+^-N:PO_4_^3−^-P ratios in the bulk (Fig. 7). Finally, NH_4_^+^-N:PO_4_^3−^-P ratios in the bulk as well as *Comamonadaceae* gen. both had a strong positive correlation with the biomass PHA-content (*p* < 0.001 for the correlation between NH_4_^+^-N:PO_4_^3—^P and the PHA content) (Fig. 7, SI Fig. A5).

### 3.4 To what extent does the reactor operation mode influence the PHA-storage response during accumulation batches?

**Fig. 8:**
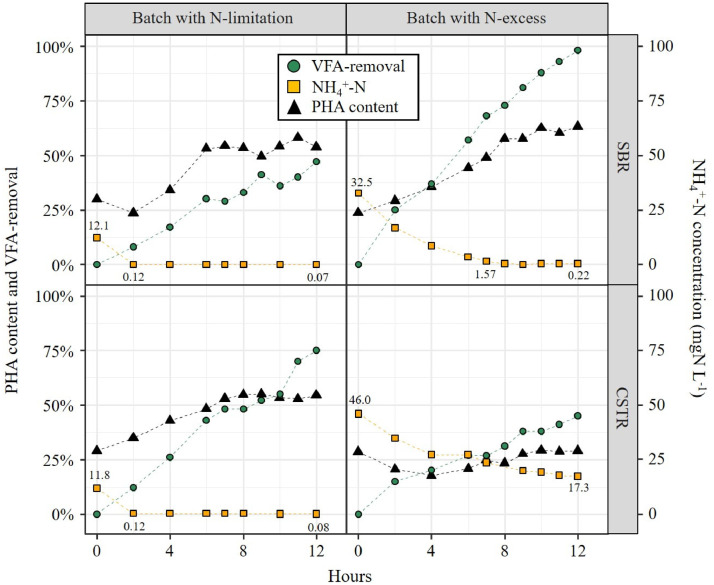
Profiles of PHA content (gCOD_PHA_ gpCOD^−1^ expressed in%), VFA-removal (in%) and NH_4_^+^-N bulk concentrations during accumulation batches with (i) predominantly N-limited conditions, and (ii) predominantly N-excess conditions. Only 2 out of 4 batches are shown for each selection reactor. The profiles for all batches are shown in SI Figure A5.

Previous results indicated a similar relationship between N availability and PHA-storage in both selection reactors. An important question is thus to what extent a similar PHA-storage response towards the availability of N is triggered in both biomasses during PHA-accumulation batches. PHA-content, NH_4_^+^-N concentrations and VFA-removal were monitored during PHA-accumulation batches (Fig. 8, SI Fig. A6). Representative examples of an accumulation batch (i) under N-limited conditions (left-hand panels) or (ii) in excess of N (right-hand panels) are shown on Fig. 8. Growth conditions were considered N-limited at NH_4_^+^-N bulk concentrations ≤ 0.1 mgN L ^−^ ^1^. The CSTR biomass accumulated PHA^2^ under N-limited conditions only, while the SBR biomass was able to accumulate PHA whether N was limiting or in excess. In contrast, NH_4_^+^-N bulk concentrations >>0.1 mgN L ^−^ ^1^ suggested growth conditions with N in excess. In the batch with N-limited conditions, both the CSTR and SBR biomasses increased their PHA content, from ∼0.30 to ∼0.55 gCOD_PHA_ gpCOD^−1^ within 6–8 h. The SBR biomass accumulated PHA under N-excess conditions also: within 7 h, the SBR biomass doubled its PHA content from 0.24 to 0.49 gCOD_PHA_ gpCOD^−1^. In contrast, the CSTR biomass did not accumulate any PHA during all 3 batches under N-excess conditions (SI Fig. A6). Overall, PHA accumulation was observed in 3 out of 4 batches for the SBR biomass, but only in 1 out of 4 batches for the CSTR biomass (SI Table A2).

The maximum PHA content observed during an accumulation batch was similar for the CSTR biomass (0.58 gCOD_PHA_ gpCOD^−1^, resp. 0.53 gPHA gVSS^−1^) compared to the SBR biomass (0.62 gCOD_PHA_ gpCOD^−1^, resp. 0.56 gPHA gVSS^−1^) (SI Table A2). The average maximum PHA content observed during the accumulation batches was 0.47 ± 0.13 gCOD_PHA_ gpCOD^−1^ (resp. 0.41 ± 0.12 gPHA gVSS^−1^) and 0.55 ± 0.08 gCOD_PHA_ gpCOD^−1^ (resp. 0.49 ± 0.08 gPHA gVSS^−1^) for the CSTR biomass and the SBR biomass, respectively. During individual batches, PHA yields up to 0.5 gCOD_PHA_ gCOD_VFA_^−1^ were observed for both CSTR and SBR biomass. On average however, PHA yields were considerably lower for the CSTR biomass (0.26 ± 0.16 gCOD_PHA_ gCOD_VFA_^−1^) compared to the SBR biomass (0.41 ± 0.14 gCOD_PHA_ gCOD_VFA_^−1^). Also, lower specific storage rates and HV:HB ratios during the batches were observed for the CSTR biomass as opposed to the SBR biomass (SI Table A2).

## 4 Discussion

### 4.1 CSTR as effective as an SBR in selecting PHA-storing biomass on a feedstock derived from MWW

The main objective of this study was to evaluate to what extent PHA-storing biomass can be successfully selected on MWW-derived feedstock in a single CSTR, as opposed to in a conventional SBR (feast/famine-based selection process). Our results demonstrate that a single CSTR can be as effective as an SBR in selecting biomass with a high PHA-storage capacity on MWW-derived feedstock. Indeed, the maximum PHA-content observed during accumulation batches was similar for the CSTR (0.58 gCOD_PHA_ gpCOD^−1^, resp. 0.53 gPHA gVSS^−1^) and the SBR biomasses (0.62 gCOD_PHA_ gpCOD^−1^, resp. 0.56 gPHA gVSS^−1^) (SI Table A2). Also, the PHA content measured in the selection CSTR indicated that the maximum storage capacity of the CSTR biomass was temporarily at least as high as 0.60–0.70 gCOD_PHA_ gpCOD^−1^, corresponding to 0.55–0.65 gPHA gVSS^−1^ (Fig. 5A). Those values are comparable to the maximum storage capacities quantified *via* accumulation tests (0.4–0.8 gPHA gVSS^−1^) for biomasses selected in SBRs on real waste-derived feedstock (Estévez-Alonso et al., 2021; Valentino et al., 2017).

Apart from selecting a biomass with a high PHA-storage capacity, the selection step should be as efficient as possible in converting C-substrate into active biomass (i.e., high substrate to biomass conversion yield). Our results demonstrate the CSTR is significantly more efficient than the SBR in terms of biomass production, with observed yields of 0.26 ± 0.09 as opposed to 0.17 ± 0.06 gCOD_Xact_ gsCOD_removed_^−1^, respectively (Fig. 4C). We hypothesize the low observed yields in the SBR resulted from the feast-famine regime. Indeed, the transient availability of (external) substrate in SBRs encourages bacteria to maximize their substrate uptake rate over their growth rate (Paul et al., 2020; Reis et al., 2011). The larger the substrate uptake rate compared to the growth rate, the more substrate is available as “excess energy” to the cell (Low and Chase, 1999). While part of the excess energy can be conserved in form of storage compounds (e.g., PHAs), the remaining excess energy is lost as heat generated by catabolic reactions (Low and Chase, 1999). Thus, by maximizing substrate uptake over growth, the amount of substrate lost through catabolic reactions increases, ultimately resulting in low substrate to biomass conversion yields. In contrast, substrate uptake rates in a CSTR are limited by the low substrate concentrations maintained in the bulk liquid, thus minimizing the imbalance between substrate uptake and growth rates. Consequently, less substrate is lost through catabolic reactions in a CSTR, and higher substrate to biomass conversion yields are in turn observed compared to in an SBR.

Overall, our results clearly demonstrate that a simple CSTR is (i) as effective as a feast-famine SBR in selecting biomass with a high PHA-storage capacity on MWW-derived feedstock, and also (ii) more efficient than the SBR in terms of C-substrate utilization. Finally, the PHA-contents observed in our selection CSTR during the last 10 days of the experiment were >0.4 gPHA gVSS^−1^, which is the identified threshold value for a commercially viable recovery of PHAs from the biomass (Bengtsson et al., 2017). Further research should investigate whether, and under which conditions, such high PHA contents can be maintained in the long-term, so that a subsequent accumulation-step can be omitted and the entire PHA production is simplified.

### 4.2 Similar microbial communities in CSTR and SBR

Our results also indicate that the similar PHA-storage capacities of the CSTR and SBR biomasses actually resulted from the selection of similar microbial communities (Fig. 3A). An important aspect to discuss is then (i) which and how many known PHA-storers figured among the dominant taxa in the CSTR and SBR selection reactors, and (ii) why similar PHA-storers have been selected in both systems.

*Comamonadaceae* gen., *Rhodocyclaceae* gen. and *Rhodobacteraceae* gen. were the dominant families in our systems (Fig. 2). Taxa belonging to those families are often reported as the dominant PHA-storers in selection SBRs fed with waste-derived feedstock (Albuquerque et al., 2013; Carvalhol et al., 2014; Chen et al., 2014; Janarthanan et al., 2016; Valentino et al., 2018). The Comamonadaceae gen., Rhodocyclaceae gen. and Rhodobacteraceae gen. observed in our study were mostly affiliated to *Comamonas* sp., *Zoogloea* sp. and an *unknown Rhodobacteraceae* sp., respectively (SI Fig. A3).Both *Comamonas* sp. and *Zoogloea* sp. are known PHA-storers (Oshiki et al., 2008). Therefore, the minimum average fraction of PHA-storers (*Comamonas* sp. + *Zooglea* sp.) was 42% and 48% in the biomass of the CSTR and SBR, respectively, during the 2nd half of the experiment when *Comamonas* sp. proliferated. The PHA-storer fraction in SBR biomass selected on real waste-derived feedstock typically ranges from 56% to 84% (Albuquerque et al., 2013; Jiang et al., 2012). Because on average >25% of the sequences could not be affiliated at the genus level, we limited ourselves to estimate a minimum fraction of PHA-storers. Therefore, one may be careful in comparing our apparent low numbers with other values reported in literature. The *Rhodobacteraceae* family – enriched in the 1st half of the experiment – contains many taxa endowed with PHA-metabolism (e.g., *Paracoccus* sp., *Amaricoccus* sp., and *Rhodobacter* sp.) (Morgan-Sagastume, 2016). Thus, the *unknown Rhodobacteraceae* sp. found in our systems could be a putative PHA-storer. However, to avoid speculation, no minimum PHA-fraction was estimated for the 1st half of the experiment.

In theory, one would expect that different microbial communities are selected in a CSTR as opposed to in the state-of-the-art SBR as a result of the different growth conditions created in each system, i.e., a constant famine vs. a feast-famine regime, respectively. Accordingly, Marang et al. (2018) observed the selection of different PHA-storers under constant famine conditions (*Zoogloea* in a continuously fed SBR) than under feast-famine conditions (*Plasticicumulans* in a pulse-fed SBR). Surprisingly, a key observation in our study was the selection of similar microbial communities – and thus PHA-storers – in the CSTR and SBR. A potential explanation might be the absence of a true feast-famine regime in our SBR, as indicated by the high feast/famine ratio (0.54 ± 0.23). In a true feast-famine regime (i.e., feast/famine ratio <0.25), a stringent famine phase ensures that the biomass undergoes a physiological adaptation where substrate uptake capacity is maximized over growth capacity (Reis et al., 2011; vanLoosdrecht et al., 1997). Consequently, microorganisms with the highest maximum substrate uptake rates have a competitive advantage during the subsequent feast phase (Reis et al., 2011). Feast/famine ratios >0.25 - such as in our study – however indicate that the famine phase is not long/stringent enough to ensure the targeted physiological adaptation (Reis et al., 2011; Valentino et al., 2017). Consequently, microbial competition might then be driven by maximum growth rate at given bulk concentrations rather than maximum substrate uptake rate (Reis et al., 2011). Since microbial competition in a CSTR is also based on maximum growth rate, and similar microbial communities were selected in both CSTR and SBR, our results highlight that when a true feast-famine regime cannot be imposed, the SBR approach creates a similar selective environment than the use of a simple CSTR. While reactor operation mode did not influence microbial competition in our study, different microbial communities were selected in the 1st and in the 2nd half of the experiment (Fig. 3B). An important question is then, what factor caused a change in selective pressure over time and therefore governed microbial competition in our systems?

### 4.3 Nutrient availability drives microbial community composition while reactor operation mode drives physiological state of the biomass

Our results indicate that the influent nutrient availability (N in particular) governed microbial selection in our systems. During our experiment and in both selection reactors, the constant increase in N availability in the influent caused significant variations of growth conditions in the two systems: from stable N-limited conditions before day 75 (1st half of the experiment) to a gradual increase in the N-availability after (2nd half of the experiment) (Fig. 6A-B). This change in growth conditions in turn resulted in a major switch in the microbial community composition (Fig. 3B). More specifically, N-limited conditions favoured the selection of *Rhodobacteraceae* gen. (*unknown* sp.), while *Comamonadaceae* gen. (*Comamonas* sp.) proliferated in the 2nd half of the experiment, as N availability increased significantly over time (Figs. 2, 6B, 7). Taxa belonging to the *Rhodobacteraceae* gen. are characterised by a high transcript abundancies of NH_4_^+^-transporters under N-limited conditions, indicative of a high affinity towards NH_4_^+^(Aanderud et al., 2019; Pfreundt et al., 2016), which could potentially explain why *Rhodobacteraceae* gen. (*unknown* sp.) were primarily selected under N-limited conditions during the 1st half of the experiment (NH_4_^+^-N < 0.1 mgN L ^−^ ^1^). In turn, representatives of the *Comamonas* sp. have a genetic predisposition to be very efficient in responding to environmental changes, notably with respect to the regulation of NH_4_^+^-N assimilation (Ma et al., 2009; Wu et al., 2018). The long-term dynamics (over several weeks) of N availability in the 2nd half of the experiment might thus have provided a competitive advantage to *Comamonas* sp., ultimately translating in the dominance of *Comamonadaceae* gen. in our systems.

Another main result of our study is that PHA-accumulation was triggered more easily for the SBR biomass than for the CSTR biomass. Indeed, PHA-accumulation by the SBR biomass was observed during most batches, independently of the N availability (SI Table A2). In contrast, PHA-accumulation in the CSTR biomass was only observed in 1 out of 4 batches, when growth was limited by the availability of N. An important question is then why the response of the SBR and CSTR biomass in terms of PHA-accumulation was different, although associated with similar microbial communities? We hypothesize the reactor operation mode had an influence on the physiological state of the biomass, ultimately with implications on the PHA accumulation response. Biomass selected in the SBR is typically in a state of internal growth limitation at the end of the cycle because microorganisms slow down synthesis of growth material (enzymes, RNA) after a prolonged absence of external C substrate (Reis et al., 2011). This internal growth limitation stimulates excessive PHA-storage during the subsequent feast-phase even though nutrients might not be limiting. An accumulation batch very much resembles the feast-phase of a regular cycle, and PHA-accumulation under nutrient excess has previously been reported for SBR biomass (Korkakaki et al., 2016a). In contrast, the CSTR biomass is continuously exposed to low substrate/nutrient concentrations and has no history with cyclic environmental changes. When transferred to the accumulation reactor and therefore exposed to high substrate/nutrient concentrations, the biomass selected in the CSTR might not be able to increase its substrate uptake rate relative to its growth rate after sudden exposure to high substrate and nutrient concentrations. If there is no relative increase of substrate uptake rate compared to growth rate during the accumulation-step, the same relative amount of C substrate will be directed towards PHA-storage than in the selection reactor, thus preventing a further increase of the PHA content. Key for triggering PHA accumulation with a biomass selected in a CSTR is therefore to impose a lower nutrient availability than the one imposed during the selection step. Since our accumulation batches were performed with a certain level of nutrients (resulting from the variable composition of the MWW-derived feedstock), the maximum PHA-storage capacity of the biomasses, especially the CSTR one, was presumably underestimated. In order to correctly evaluate the maximum PHA-storage capacity of CSTR biomass in particular, accumulation batches should be performed under starvation of a nutrient, e.g., N or P. Finally, a main weakness of our study is the lack of accumulation batches during the 1st half of the experiment when growth was N-limited. Consequently, our results do not allow to conclude on the extent to which selection was successful under N-limited conditions.

### 4.4 CSTR can select PHA-storing biomass in absence of P or N limitation

So far, the selection of biomass with high PHA-storage capacity in a single CSTR (up to 0.77 gPHA gVSS^−1^ and >90% PHA-storers) has been studied under P limitation only, and using acetate/propionate as sole C-substrates (Brison et al., 2022a; Cavaille et al., 2016). Our results demonstrate for the first time that a single CSTR can be successfully used for the selection of a biomass with a high PHA-storage capacity also in the absence of P-limitation and on real MWW-derived feedstock, i.e., on a more complex C substrate composition. In particular, the highest PHA-storage capacity of our CSTR biomass (up to 0.70 gCOD_PHA_ gpCOD^−1^, resp. 0.65 gPHA gVSS^−1^) was observed in the 2nd half of our experiment, when neither P nor N were limiting growth (Figs. 5A and 6B-C). An important question is then, what were the potential drivers for this selection? Several possible mechanisms could explain the selection of PHA-storers in absence of N or P limitation: (i) dynamic environmental growth conditions (Pei et al., 2022; Reis et al., 2011; vanLoosdrecht et al., 1997), (ii) the limited availability of a nutrient other than N or P, such as iron, sulfur, etc. (Steinbuchel and Schlegel, 1989; Thingstad et al., 2005), and (iii) the composition of the feedstock in terms of organic substrates, i.e., the large fraction of VFAs in the influent (Korkakaki et al., 2016b; Pei et al., 2022).

Dynamic environmental growth conditions – such as in selection SBRs or in WWTPs – are known to provide a competitive advantage to PHA-storers (Pei et al., 2022; Reis et al., 2011; vanLoosdrecht et al., 1997). In the feast-famine SBR approach, transient availability of C-substrate during the cycle favours the selection of PHA-storers (Reis et al., 2011). In full-scale WWTPs, the development of ecological niches favorable to PHA-storers results from the sequential exposure of the biomass to changing C-substrate availability and/or redox conditions along the treatment chain (Pei et al., 2022; vanLoosdrecht et al., 1997). In both the selection SBRs or the WWTPs, the characteristic time of the “dynamic growth conditions” is therefore few hours. However, the CSTR biomass in our study was neither exposed to a transient availability of C-substrate nor to changing redox conditions over such short periods. Instead, a gradual increase in the N availability was observed over several days/weeks, and it is unclear if/how such conditions could provide a competitive advantage to PHA-storers.

Another mechanism that could have triggered the selection of PHA-storers is a limitation of a nutrient other than N and P. Increasing cell size/surface through storage of C-substrate (e.g., in form of PHA) allows bacteria to maintain high diffusive transport across the cell membrane and, ultimately, to improve their affinity towards a limiting nutrient (Ovreas et al., 2003; Thingstad et al., 2005). Therefore, a competitive advantage for PHA-storers could have resulted from any other nutrient limitation that triggers PHA-storage, such as sulfate, potassium, magnesium or iron limitation (Steinbuchel and Schlegel, 1989). Unfortunately, our experimental design did not allow to pin-point if, and which, specific nutrients might have been limiting growth in the 2nd half of the experiment.

Finally, a key aspect for the selection of PHA-storers is the composition of the C-substrate. PHA-storage requires readily biodegradable C-substrate. Therefore, the higher the fraction of readily biodegradable C-substrate in the influent, the more PHA-storage can be used as a competitive advantage by storing organisms. In WWTPs, primary treatment removes slowly biodegradable particulate C-substrate from the MWW and in turn increases the fraction of readily biodegradable C-substrate in the influent to biological treatment. Accordingly, full-scale WWTPs equipped with primary treatment are associated with a higher fraction of PHA-storers than WWTPs without (Pei et al., 2022). Our MWW-derived feedstock consisted of ∼80% readily biodegradable C-substrate in form of VFAs, which serve as direct pre-cursors to PHAs and are the preferred substrate for microbial PHA-storage (Korkakaki et al., 2016b; Pei et al., 2022). Overall, our results thus suggest that the use of MWW-derived feedstock where VFAs represent a major fraction of the C-substrate might be sufficient for the selection of a biomass with a high PHA-storage capacity in a CSTR. Yet, Brison et al. (2022a) demonstrated that selection of PHA-storers on such favourable C-substrate composition can be enhanced *via* P-limitation, i.e., nutrient limitation. Future research should now aim to better understand (i) which nutrients (N, P, iron, sulfate, etc.) might be naturally limiting in MWW-derived feedstock, and (ii) to what extent the limited availability of such nutrients (other than P) benefits the selection process.

### 4.5 Practical implications

Our results demonstrate that dynamics in the nutrient availability of MWW-derived feedstock may result in significant changes in growth conditions (e.g., from N-limited to N-excess), which in turn can cause a major switch of the microbial community composition and ultimately of PHA storage capacity. A robust selection process should however be able to maintain a stable microbial community over time in order to ensure a steady PHA-storage capacity, a key aspect for practice. An important question is therefore how to avoid fluctuations in the feedstock composition, to better control the selection process over long term. In our study, the VFA-rich feedstock was derived from the fermentation of primary sludge only (from a primary clarifier). The fermentation of primary sludge from primary clarifiers or micro-sieves results in the production of a fermented broth associated with a low nitrogen availability (Brison et al., 2022b). In practice however, a mixture of primary sludge and excess sludge from biological processes will be directed towards anaerobic fermentation (Brison et al., 2022b). As opposed to the fermentation of primary sludge only (Brison et al., 2022b), the fermentation of a blend excess sludge results in a VFA-rich feedstock with a much higher N and P availability (Ucisik and Henze, 2008; Yuan et al., 2010). We therefore expect that the fermentation of a blend sludge should result in a more steady excess of N and P in the selection reactor, thus preventing shifts of microbial communities as a result of fluctuating growth conditions. Further, especially growth conditions with N and P excess led to a high PHA content of 0.70 gCOD_PHA_ gpCOD^−1^ in the selection CSTR. We therefore suggest that not only a successful and robust selection but even the production of PHA in single CSTR might be possible under real conditions.

Yet, until it has been demonstrated that a PHA content >0.4 gPHA gVSS^−1^ can be consistently maintained over long-term in a selection CSTR, PHA production will rely on an accumulation step following the selection step. In that context, our results suggest the CSTR biomass must be exposed to stringent nutrient limitation/starvation to achieve successful PHA-accumulation. Therefore, industrial wastewaters containing too little nutrients to sustain growth in a selection reactor should be considered as feedstock for the PHA-accumulation step (Tamis et al., 2014, 2018). This is for example the case for effluents from potato-starch factory (Morgan-Sagastume et al., 2020), sugar factory (Anterrieu et al., 2014), brewery (Ince et al., 2000), or palm oil mill (Chin et al., 1996).^3^ In return, MWW-derived feedstock could then be entirely directed towards the selection-step.

## 5 Conclusions

• A biomass with high PHA-storage capacity of up to 0.70 gCOD_PHA_ gpCOD^−1^ (resp. 0.65 gPHA gVSS^−1^) was successfully selected in a CSTR fed with MWW-derived feedstock. The storage capacity of the biomass selected in such a system was similar to the one of the biomass selected in a state-of-the-art feast-famine SBR. Yet, the simple CSTR was 50% more efficient than the SBR in terms of substrate to biomass conversion yields.
• The CSTR selected biomass with high PHA-storage capacity in absence of P- and N- limitations. Meanwhile, the exact mechanisms driving this selection remain unidentified but could potentially result from (i) the limitation of a nutrient other than N or P, and/or (ii) a favourable influent composition in terms of C-substrate, i.e., rich in VFAs.
• Nutrient availability combined with the absence of a true feast-famine regime in theSBR drove the microbial community composition. As a consequence, similar microbial communities were selected in the CSTR and in the SBR. Stable N limiting conditions resulted in a microbial community where *Rhodobacteraceae* gen. was the most abundant taxon. In contrast, dynamic N-excess conditions coincided with the selection of the known PHA-storer *Comamonas*.
• Reactor operation mode however influences the physiological state of the biomass and, ultimately, the PHA-storage response during accumulation batches. PHA-accumulation in the CSTR biomass was triggered only when N was fully removed from the bulk while PHA-accumulation in the SBR biomass occurred also with N in excess.

## Declaration of Competing Interest

The authors declare that they have no known competing financial interests or personal relationships that could have appeared to influence the work reported in this paper.

## Data Availability

Data is provided on an online repository and can be found under the DOI provided in the manuscript

## Appendix Supplementary materials

Image, application 1

## Footnotes

1 COD:N/COD:P ratios at which growth can start to be N/P-limited. The values were estimated by assuming typical cellular N and P contents of 85 mgN gCOD_cell_^-1^ and 14 mgP gCOD_cell_^-1^ and substrate to biomass conversion yields of 0.5 gCOD_cell_ gCOD_substrate_^-1^.

2 “Accumulating PHA” is used here to describe an increase of the biomass PHA-content during the batch.

3 assuming threshold values of 25gCOD gN^-1^ and 140 gCOD gP^-1^ above which cell growth might be nutrient-limited - *Those threshold values were estimated by assuming typical cellular N and P contents of 85 mgN gCODcell^-1^ and 14 mgP gCODcell^-1^*Tchobanoglous, G., L. B. Burton, R. Tsuchihashi and H. D. Stensel (2013)*. Wastewater engineering: Treatment and Resource Recovery. New York, NY, McGraw-Hill Professional ,and substrate to biomass conversion yields of 0.5 gCODcell gCODsubstrate^-1^*
