# Supplementary material for: Single CSTR can be as effective as an SBR in selecting PHA-storing biomass from municipal wastewater-derived feedstock
Source: Water Res X. 2023 Jan 10;18:100165. doi: 10.1016/j.wroa.2023.100165 (PMC10214291; doi:10.1016/j.wroa.2023.100165)
Supplement: Supplementary file 1 [file mmc1.docx]

Single CSTR can be as effective as an SBR in selecting PHA-storing biomass from municipal wastewater-derived feedstock

**SUPPLEMENTARY INFORMATION A**

Antoine Brison*,**, Pierre Rossi***, Nicolas Derlon*

* Eawag, Swiss Federal Institute of Aquatic Science and Technology, 8600 Dübendorf, Switzerland
** ETH Zürich, Institute of Environmental Engineering, 8093 Zürich, Switzerland
*** Central Environmental Laboratory, School of Architecture, Civil and Environmental Engineering, Ecole Polytechnique Fédérale de Lausanne Lausanne, Switzerland

Emails of the authors: [Antoine.brison@eawag.ch](mailto:Antoine.brison@eawag.ch)

[Pierre.rossi@epfl.ch](mailto:Pierre.rossi@epfl.ch)

[Nicolas.derlon@eawag.ch](mailto:Nicolas.derlon@eawag.ch)

Corresponding author: [Nicolas.derlon@eawag.ch](mailto:Nicolas.derlon@eawag.ch)

# Material and methods

## Reactor operation

| 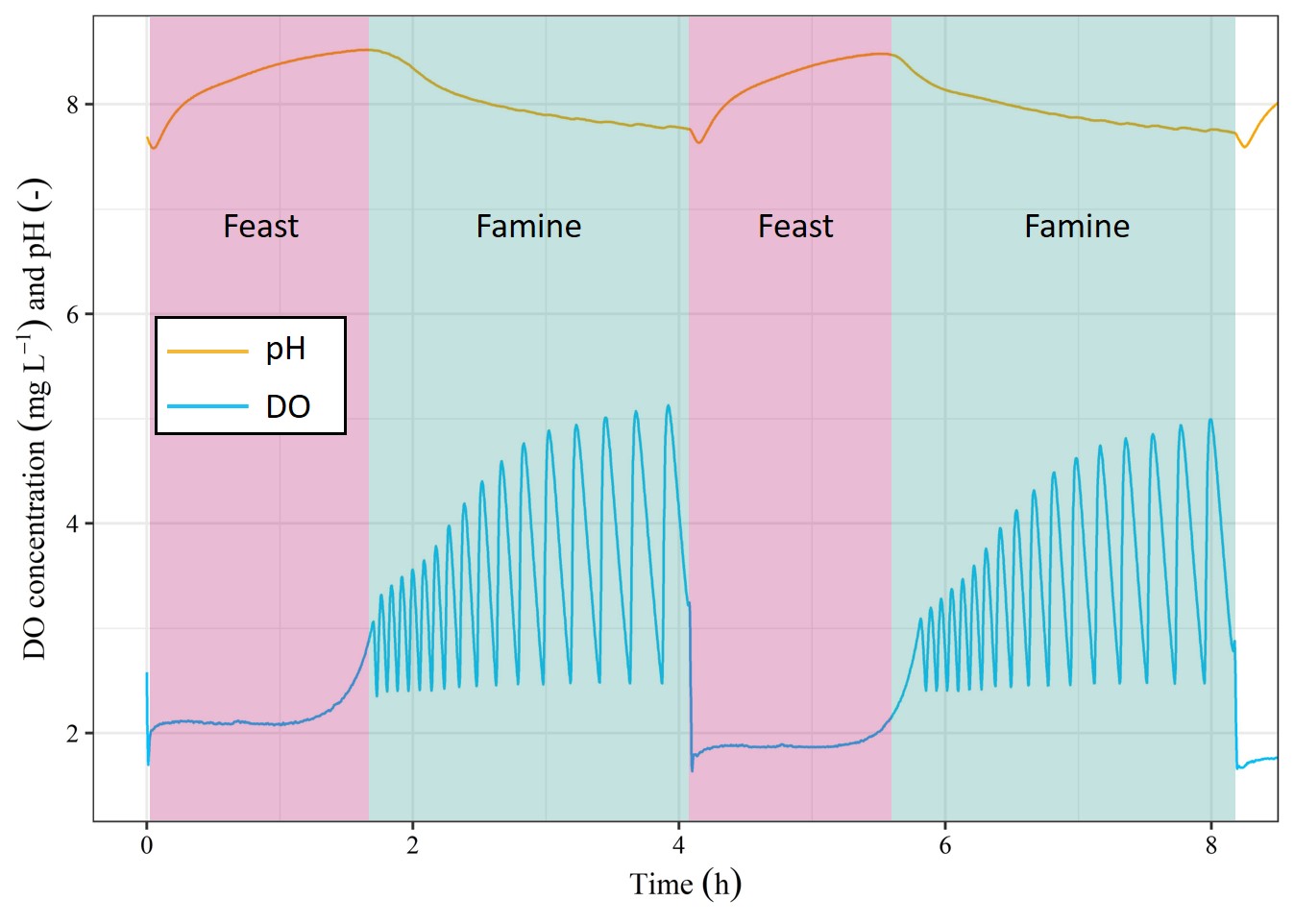  **SI Figure A1:** Typical observed dissolved oxygen (DO) concentration and pH profiles during operation of the selection SBR. The end of the feast phase was determined as the point in time where the pH stoped increasing and/or DO concentration started to drastically increase, i.e., after complete uptake of VFAs. |
| --- |

## PHA extraction, hydrolysis and analysis

Roughly 20 mg of lyophilised solids were mixed in a glass vial with 1 mL of acidified methanol (20% H_2_SO_4_) and 1 mL of chloroform containing 1 mg mL^-1^ of heptadecane (Sigma-Aldrich, Germany) as internal standard. Samples were then incubated at 100°C for 3.5 hours for PHA extraction and further hydrolysed into its monomers. Samples were then cooled down on ice and vortexed for 1 minute after adding 0.5 mL of nanopure water to aid phase separation. The lower phase (containing the chloroform) was carefully pipetted and transferred into 3 mL vials prior to analysis with a gas chromatograph coupled to a flame ionization detector (GC-FID) (Trace 1300 GC, Thermo Scientific, USA) and equipped with Zebron ZB-WAXplus (60 m x 0.53 mm x 1.00 µm) and Z-Guard (10 m x 0.32 mm) columns (both Phenomenex, USA). The detailed instrument method can be found in Lanham et al. (2013).

## DNA extraction

200 μL of the homogenized biomass were mixed with 400 μL of TE buffer (10 mM Tris-HCl, 1 mM EDTA-Na_2_ pH 8.0) and 100 μL of lysozyme solution (25 mg mL^−1^), prior to incubation for 1 hour at 37 °C. DNA was extracted using the Maxwell® 16 robot and dedicated Tissue DNA purification kits (all Promega, USA) according to the manufacturer instructions. Quality measurement and quantification of the extracted DNA samples were assessed with agarose gels and fluorometric assays (Qubit ver. 2.0, Life Technologies, USA), respectively.

# Results

## Microbial community analysis

| 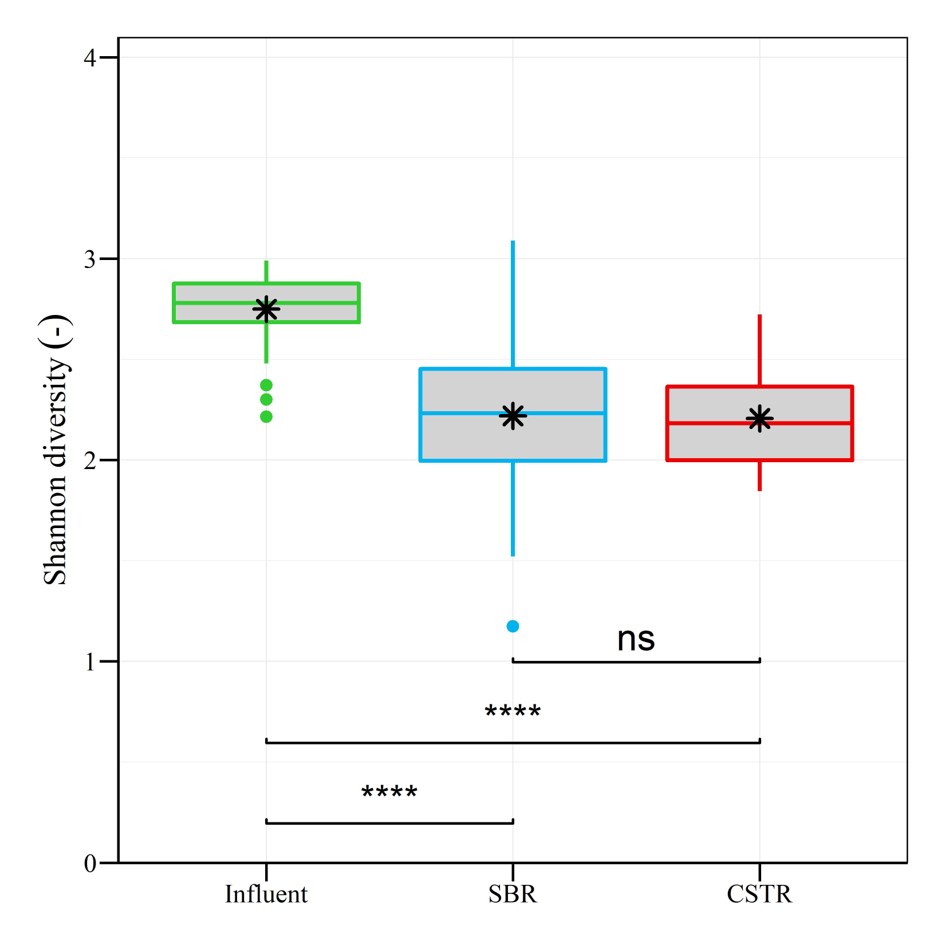  SI Figure A2: Boxplot showing the Shannon-Weaver diversity of the microbial communities at the Family taxonomic level in the influent and in both selection reactors. The black stars in the boxes are the mean values. The symbols on top of the brackets indicate the statistical significance of the difference between mean values according to an Independent Samples t-test: ns (not significant, p≥0.05), * (p<0.05), ** (p<0.01), *** (p<0.001) and **** (p<0.0001). |
| --- |

| 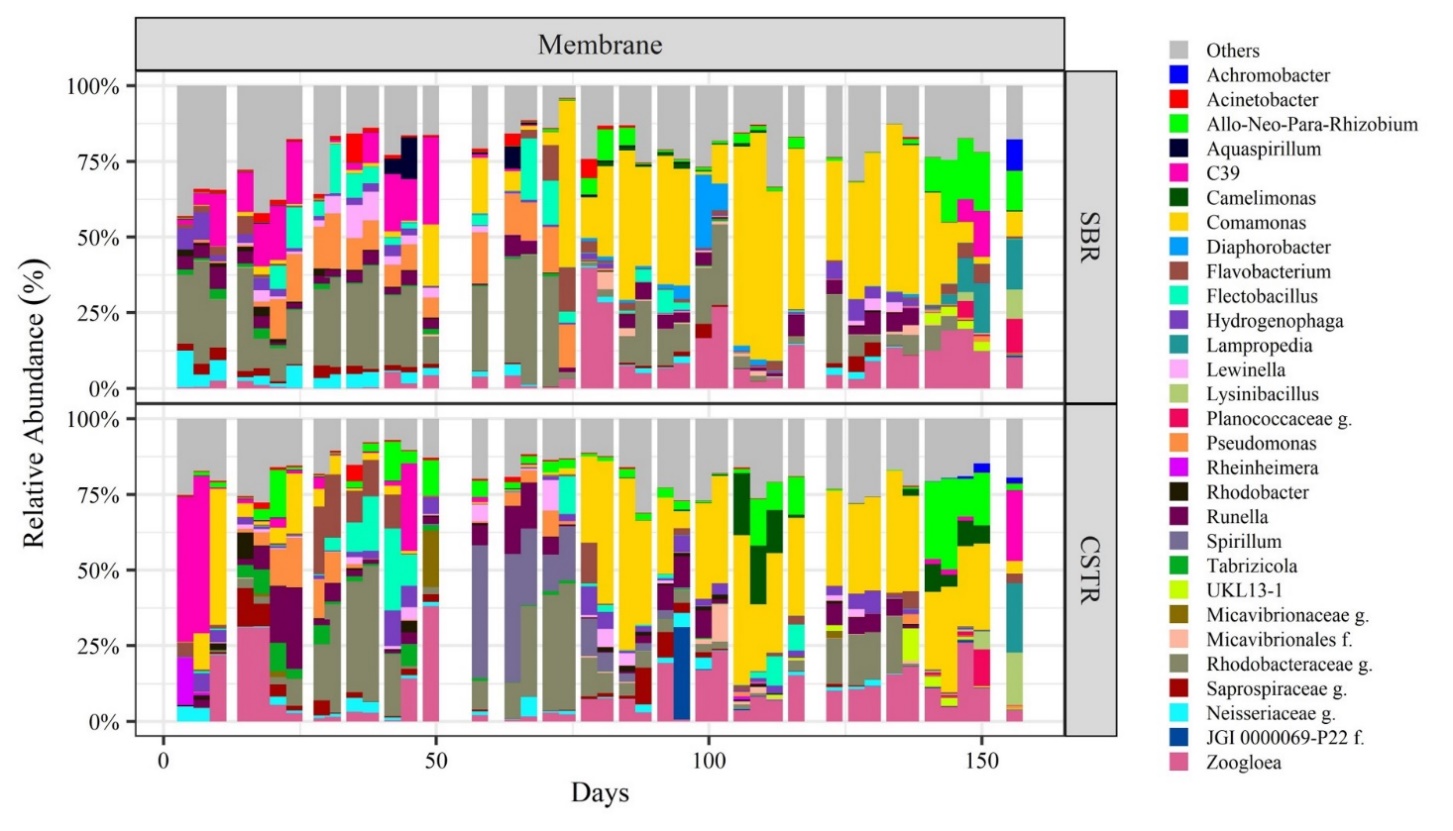  SI Figure A3: Microbial community composition over time in both selection reactors at the Genus taxonomic level. The group “Others” contains all taxa with a maximum relative abundance <8% with respect to all samples. . |
| --- |

## Link between influent composition, stoichiometric growth conditions, microbial community composition, and PHA-storage

| 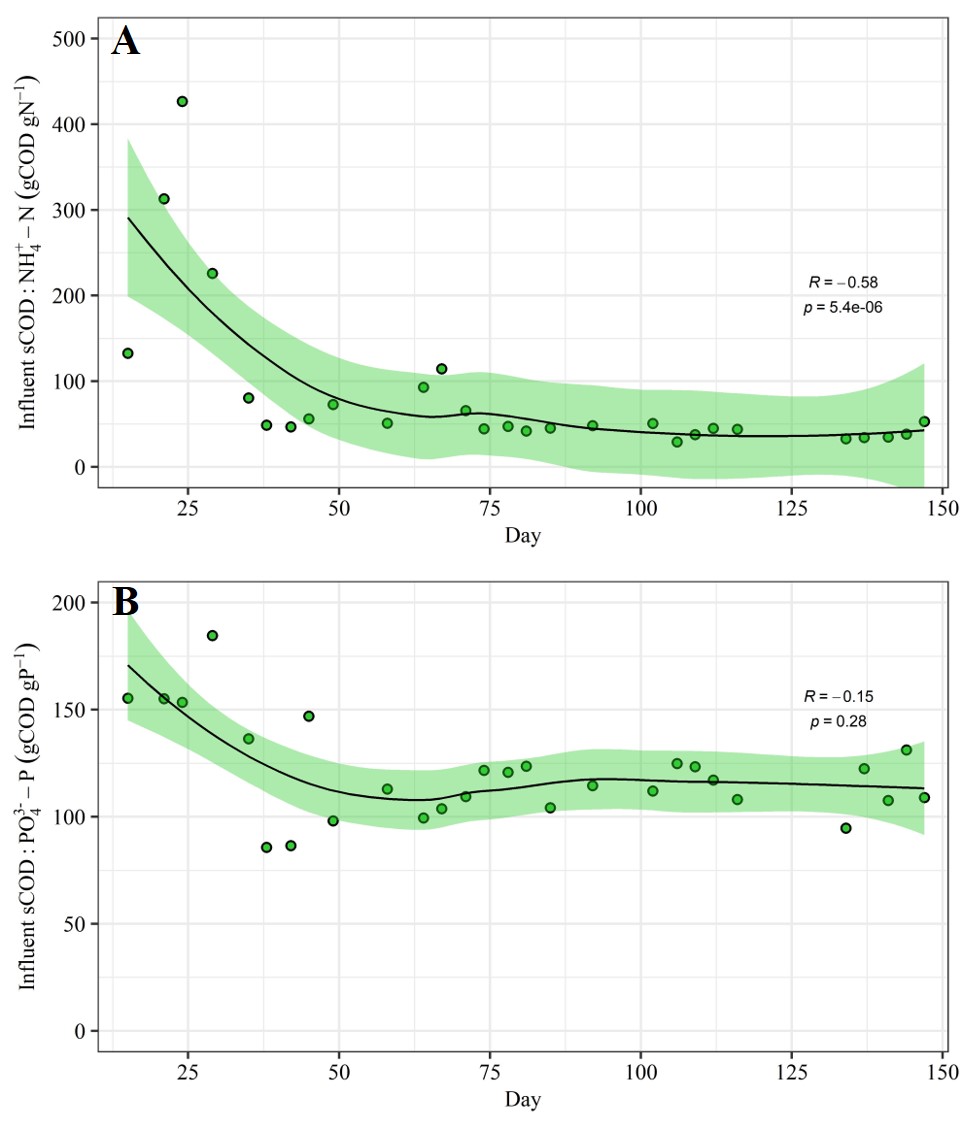  SI Figure A4: Time series of (A) sCOD:NH_4_^+^-N ratio and (B) sCOD:PO_4_^3-^-P ratio in the influent. Local regression fitting (LOESS) (black line) and 95% confidence interval (shaded area) were added to help visualize general trends. The Kendall’s tau coefficient (R) indicates whether the variable of interest followed a strong monotonic upward (R>0.3) or downward (R<-0.3) trend over time. The p-value (p) indicates the statistical significance of the observed trend. Only p-values < 0.05 were considered statistically significant. |
| --- |

| 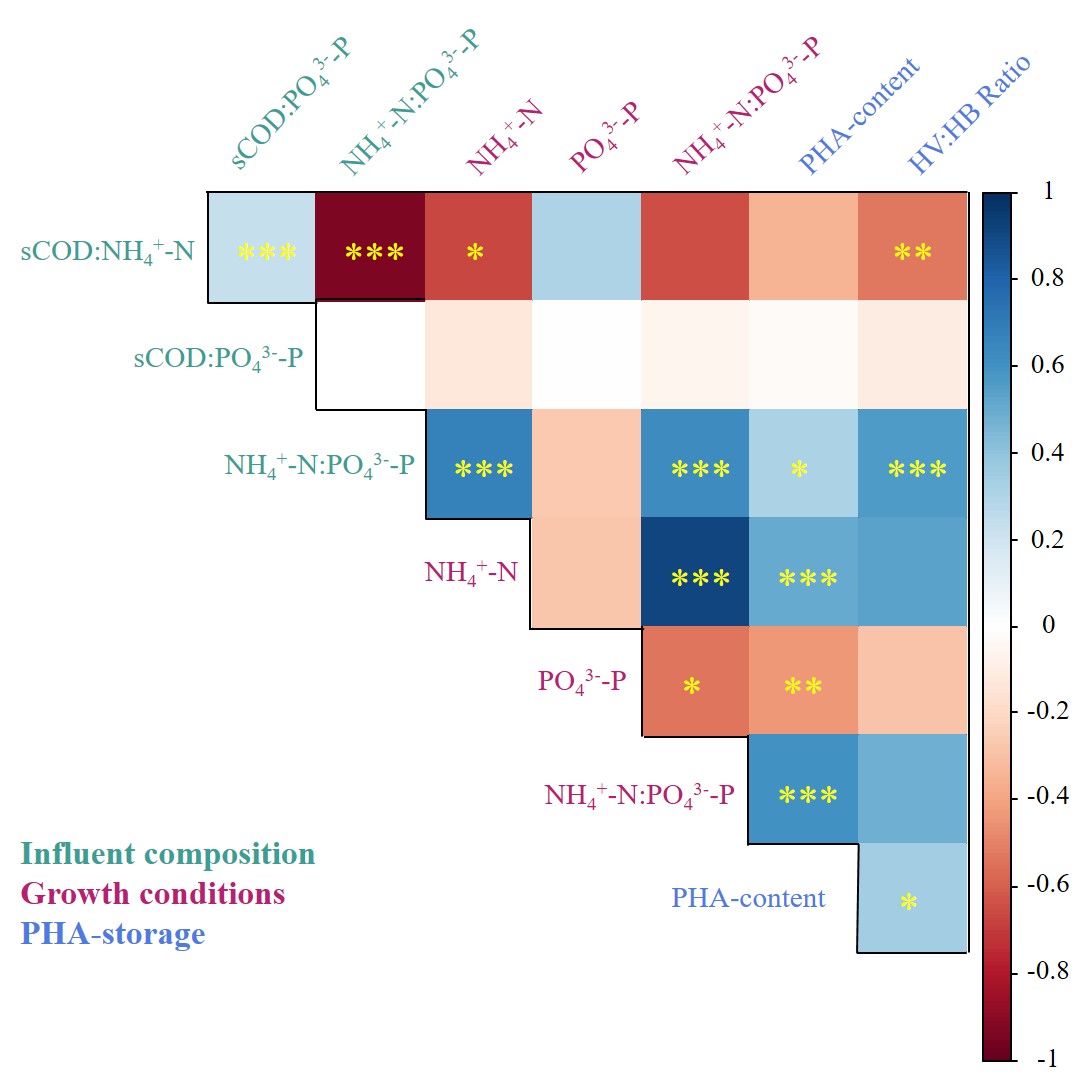  SI Figure A5: Heatmap based on spearman-ranked correlation between the different environmental variables. The yellow stars show the p-values of the calculated rho coefficients: * p-value <0.05, ** p-value <0.01, *** p-value < 0.001. |
| --- |

**SI Table A1:** Spearman correlation matrix between bacterial taxa at the Family taxonomic level and environmental variables. Only statistically significant rho-coefficients with p-values < 0.05 are shown.

| **Bacterial taxa** | **Influent composition** | | | **Growth conditions** | | | **PHA-storage** | |
| --- | --- | --- | --- | --- | --- | --- | --- | --- |
|  | sCOD:NH_4_^+^-N | sCOD:PO_4_^3-^-P | NH_4_^+^-N: PO_4_^3-^-P | NH_4_^+^-N | PO_4_^3-^-P | NH_4_^+^-N: PO_4_^3-^-P | PHA content | HV:HB ratio |
| *Beijerinckiaceae* | -0.62 | NA | 0.64 | 0.63 | -0.45 | 0.70 | 0.57 | 0.51 |
| *Chitinophagaceae* | -0.42 | NA | 0.44 | 0.43 | NA | 0.32 | NA | 0.54 |
| ***Comamonadaceae*** | -0.77 | NA | 0.72 | 0.61 | -0.38 | 0.66 | 0.46 | 0.55 |
| *Flavobacteriaceae* | NA | NA | NA | NA | NA | NA | NA | NA |
| *Hydrogenophilaceae* | NA | NA | NA | NA | NA | NA | NA | NA |
| *Hyphomonadaceae* | NA | NA | NA | NA | NA | 0.34 | 0.35 | NA |
| *Prevotellaceae* | 0.46 | NA | -0.51 | -0.54 | NA | -0.58 | NA | NA |
| *Pseudomonadaceae* | 0.57 | 0.29 | -0.51 | -0.37 | 0.44 | -0.44 | NA | -0.51 |
| *Rhizobiaceae* | -0.32 | NA | 0.32 | NA | -0.58 | 0.38 | 0.31 | NA |
| ***Rhodobacteraceae*** | 0.56 | NA | -0.59 | -0.57 | 0.46 | -0.64 | -0.60 | -0.58 |
| ***Rhodocyclaceae*** | NA | NA | NA | 0.32 | NA | 0.31 | NA | 0.52 |
| *Saprospiraceae* | 0.47 | NA | -0.44 | -0.49 | 0.44 | -0.55 | -0.51 | NA |
| *Spirosomaceae* | 0.50 | NA | -0.55 | -0.59 | NA | -0.53 | -0.48 | -0.54 |

## Accumulation batches

**SI Table A2:** Table summarizing initial conditions and performances of the different accumulation tests performed.

|  | Units | Batch 1 | | Batch 2 | | Batch 3 | | Batch 4 | |
| --- | --- | --- | --- | --- | --- | --- | --- | --- | --- |
|  |  | Day 108 | | Day 115 | | Day 136 | | Day 143 | |
|  |  | SBR | CSTR | SBR | CSTR | SBR | CSTR | SBR | CSTR |
| Initial conditions  (Time = 0) |  |  |  |  |  |  |  |  |  |
| sCOD concentration | [mgCOD L^-1^] | 2853 | 3476 | 2638 | 2850 | 4516 | 4664 | 5966 | 7438 |
| VFA concentration* | [mgCOD L^-1^] | 2616 | 3150 | 2277 | 2521 | 4358 | 4208 | 5831 | 6957 |
| VFA loading** | [gCOD gpCOD^-1^] | 2.6 | 3.8 | 3.0 | 2.7 | 3.4 | 2.8 | 4.1 | 4.2 |
| VFA:NH_4_^+^-N | [gCOD gN^-1^] | 102 | 98 | 107 | 118 | 58 | 48 | 93 | 104 |
| VFA: PO_4_^3-^-P | [gCOD gP^-1^] | 324 | 384 | 244 | 274 | 285 | 269 | 382 | 471 |
| PHA content | [gCOD_PHA_ gpCOD^-1^] | 0.24 | 0.29 | 0.30 | 0.29 | 0.48 | 0.65 | 0.44 | 0.58 |
| Performance parameters |  |  |  |  |  |  |  |  |  |
| Maximum PHA content*** | [gCOD_PHA_ gpCOD^-1^] | 0.62 | 0.29 | 0.54 | 0.54 | 0.61 | 0.58 | 0.44 | 0.47 |
|  | [gPHA gVSS^-1^] | 0.56 | 0.25 | 0.47 | 0.48 | 0.55 | 0.53 | 0.38 | 0.41 |
| PHA yield | [gCOD_PHA_ gCOD_VFA_^-1^] | 0.5 | 0.13 | 0.48 | 0.47 | 0.46 | 0.3 | 0.21 | 0.15 |
| HV:HB ratio | [-] | 2.45 | 0.55 | 2.83 | 2.5 | 2.84 | 0.72 | 1.62 | 2.43 |
| qSTOR**** | [gCOD_PHA_ gCOD_Xact_ ^-1^ h^-1^] | 3.01 | 0.45 | 1.92 | 2.41 | 4.00 | 1.84 | 1.24 | 0.96 |
| N availability |  | N excess | N excess | N limited | N limited | N excess | N excess | N excess | N excess |
| * Consisting mainly of propionate since pure propionate was added to the MWW-derived feedstock. ** Loading with respect to the total biomass (as pCOD including PHA) at the beginning of the batch.  *** Average-values once PHA content stabilized. For the CSTR Batch 3 and 4, the “maximum” PHA content is lower than the initial, meaning that growth was favoured over storage during the batch.  **** The specific storage rate was calculated as: $\text{qSTOR}\mathbf{=}\frac{\left( \text{pCOD}_{\text{PHA}\text{,}\text{End}}\text{-}\text{pCOD}_{\text{PHA}\text{,}\text{Start}} \right)}{\text{pCOD}_{\text{Xact}}\text{∙∆}\text{t}}$, with pCOD_PHA,Start_ the PHA concentration (gCOD L^-1^) at the start of the batch, pCOD_PHA,End_  the PHA concentration (gCOD L^-1^) at the point in time when the PHA content stabilized, Δt the time period (h) from the start of the batch until the PHA content stabilizes, and pCOD_Xact_ the average concentration of active biomass (gCOD L^-1^) during Δt. | | | | | | | | | |

| 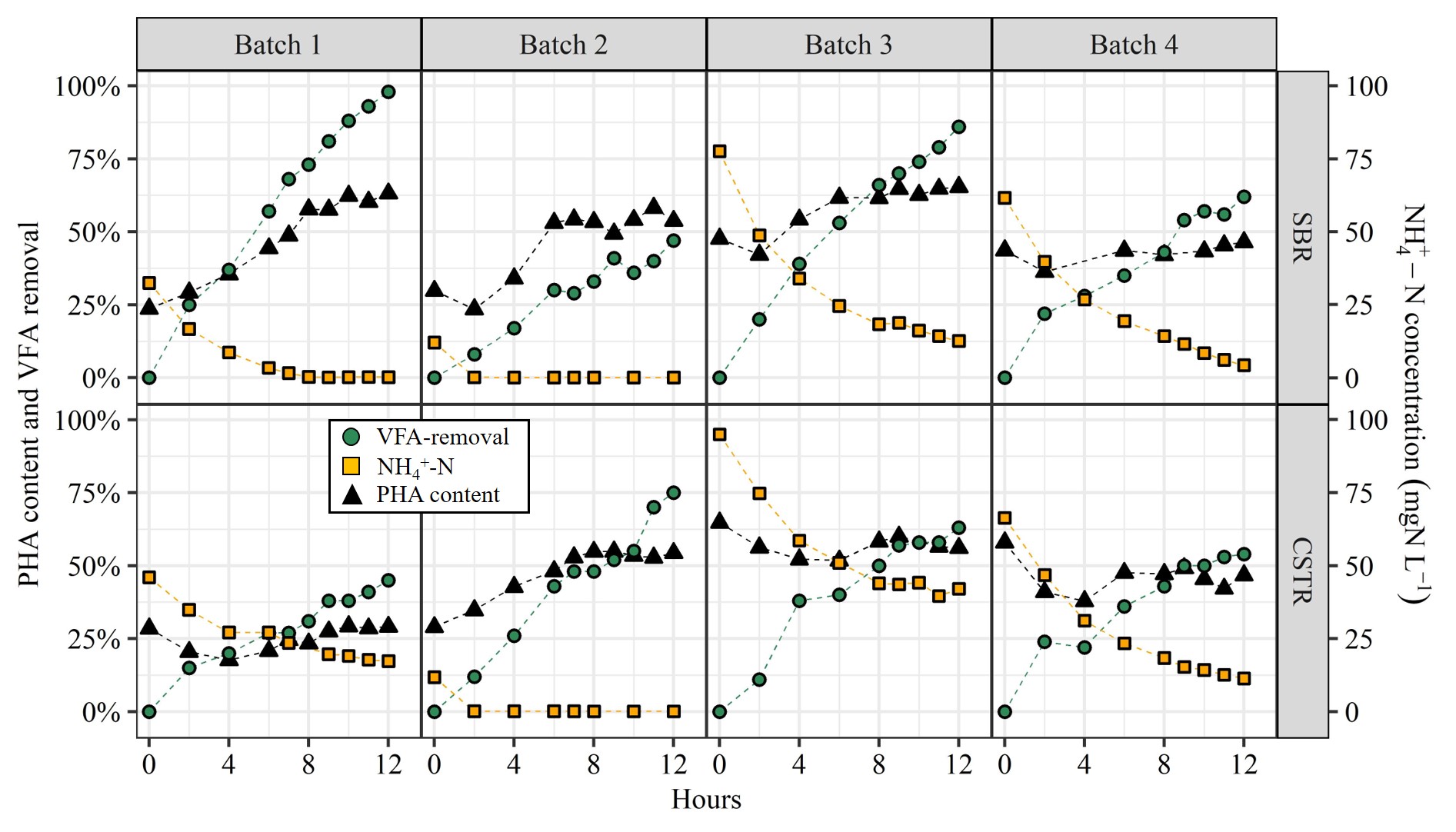  SI Figure A6: Profiles of PHA content (gCOD_PHA_ gpCOD^-1^ expressed in %), VFA-removal (in %) and NH_4_^+^-N bulk concentrations during accumulation batches. |
| --- |

# References

Lanham, A.B., Ricardo, A.R., Albuquerque, M.G.E., Pardelha, F., Carvalheira, M., Coma, M., Fradinho, J., Carvalho, G., Oehmen, A. and Reis, M.A.M. (2013) Determination of the extraction kinetics for the quantification of polyhydroxyalkanoate monomers in mixed microbial systems. Process Biochemistry 48(11), 1626-1634.

Layer, M., Adler, A., Reynaert, E., Hernandez, A., Pagni, M., Morgenroth, E., Holliger, C. and Derlon, N. (2019) Organic substrate diffusibility governs microbial community composition, nutrient removal performance and kinetics of granulation of aerobic granular sludge. Water Research X 4.
